# Acromegaly and Cushing's syndrome caused by a neuroendocrine tumor arising within a sacrococcygeal teratoma

**DOI:** 10.1002/ccr3.1148

**Published:** 2017-09-14

**Authors:** Tarig Babiker, Efstathia Kyrodimou, Daniel M. Berney, Mark Gurnell, William M. Drake, Antonia Brooke

**Affiliations:** ^1^ Departments of Endocrinology and Histopathology Royal Devon and Exeter Hospital Barrack Road Exeter EX2 5DW UK; ^2^ Department of Histopathology Barts Cancer Institute Queen Mary, University of London London EC1A7BE UK; ^3^ Metabolic Research Laboratories Wellcome Trust‐MRC Institute of Metabolic Science National Institute for Health Research Cambridge Biomedical Research Centre Addenbrooke's Hospital University of Cambridge Cambridge CB2 0QQ UK; ^4^ Department of Endocrinology St Bartholomew's Hospital London EC1A7BE UK

**Keywords:** Acromegaly, ACTH, Cushing's, neuroendocrine, teratoma

## Abstract

A 60‐year‐old man with a pre‐existing stable sacrococcygeal teratoma developed acromegaly, ectopic Cushing's syndrome, and 5HIAA secretion. To our knowledge, this represents the first reported case of ACTH and serotonin secretion, and likely GHRH or GH cosecretion, from a sacrococcygeal teratoma in an adult.

## Case Description

**Figure 1:**
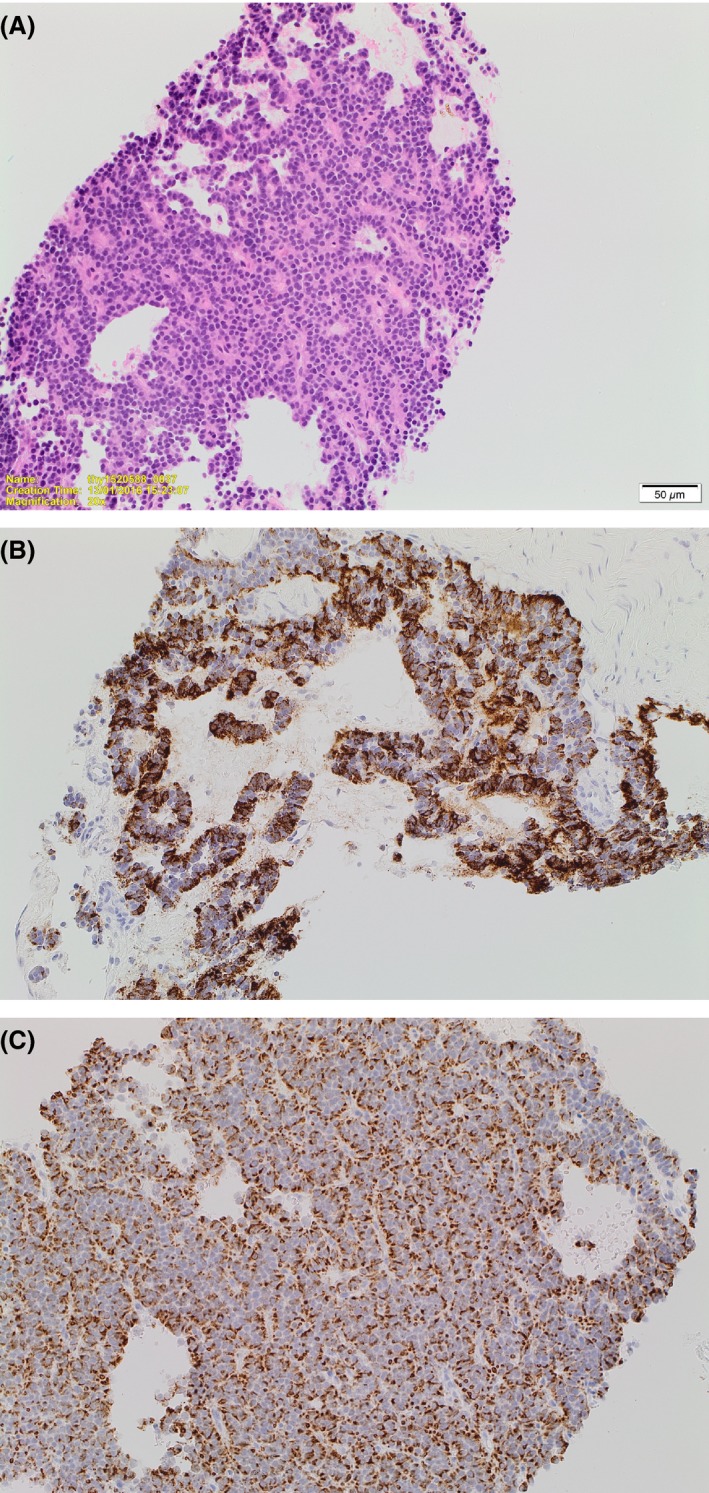
Histology of the trucut biopsy. (A) H&E showing a core of well‐differentiated neuroendocrine tumor with bland ovoid nuclei arranged in a partially cribriforming and partly solid architecture. (B) Immunochemistry showing strong positivity for ACTH in the vast majority of cells. (C) Immunochemistry for CAM 5.2 showing a perinuclear dot positivity, typical for neuroendocrine tumors.

A 60‐year‐old man presented with clinical features of acromegaly. He had a background of type 1 diabetes, diagnosed aged 28 years, hypertension and a C3–C6 laminoplasty aged 54 years for cervical stenosis. He had continued surveillance following an exploration and partial resection of a sacral dermoid cyst aged 8 years. CT imaging showed a complex, partly cystic, partly solid presacral mass with some erosion of the lower sacrum and coccyx (Fig. 1). The cystic component had required intermittent radiological‐guided drainage to relieve abdominal pain.

Endocrine testing confirmed growth hormone (GH) excess (nadir serum GH after 75 g oral glucose 9.8 mcg/L; serum IGF‐1 72.4 nmol/L (reference range 9.0–40.0)). The pituitary gland was homogenous on T1‐weighted magnetic resonance imaging (MRI), with no focal mass lesion, although with a convex superior margin. Repeat pituitary imaging with T1‐ and T2‐weighted and T1 dynamic sequences showed no clear focal abnormality, but persistent convexity of the superior aspect of the gland. Due to the absence of a definite surgical target, he was initially treated medically. However, dopamine agonist and somatostatin analogue therapy were poorly tolerated due to unpredictable hypoglycemic events and gastrointestinal side effects and, after a period of time, medical therapy was stopped.

^11^C‐methionine PET‐CT coregistered with volumetric MRI demonstrated diffuse hyperintensity within the pituitary, but no discrete lesion. Somatostatin receptor scintigraphy was reported to show physiological uptake in the bladder and physiological uptake in the pituitary sella. He underwent pituitary exploration and partial transphenoidal hypophysectomy; histology revealed normal pituitary tissue only, but 3 months postoperatively his serum IGF‐1 was just 11 nmol/L (age‐related reference range 5–26 nmol/L) and has remained normal thereafter. Anterior pituitary function was otherwise normal postoperatively.

In the weeks preceeding surgery, he developed peripheral edema. Within weeks of surgery, he was admitted with severe fluid retention and abdominal bloating. He had gained 15 kg in weight with persistent hypokalemia despite the use of potassium‐sparing diuretics (amiloride, spironolactone). There was no proteinuria, and serum albumin was 30 g/L and he had normal cardiac function as determined by MRI. A pulmonary embolus was identified on CT pulmonary angiogram but deemed not large enough to cause cardiac strain, and there was no associated inferior vena cava obstruction to explain his edema. He suffered recurrent infections: bilateral pneumonia, cellulitis, influenza, and norovirus‐associated gastroenteritis. Cortisol excess was suspected and confirmed on a low‐dose dexamethasone suppression test (cortisol postdexamethasone 1070 nmol/L) and an elevated midnight cortisol (>1750 nmol/L). ACTH was elevated (188 ng/L; reference range 0–40 ng/L); with all tests performed after resolution of his acute illness. Postoperative cortisol ranged between 800 and 1728 nmol/L.

**Figure 2:**
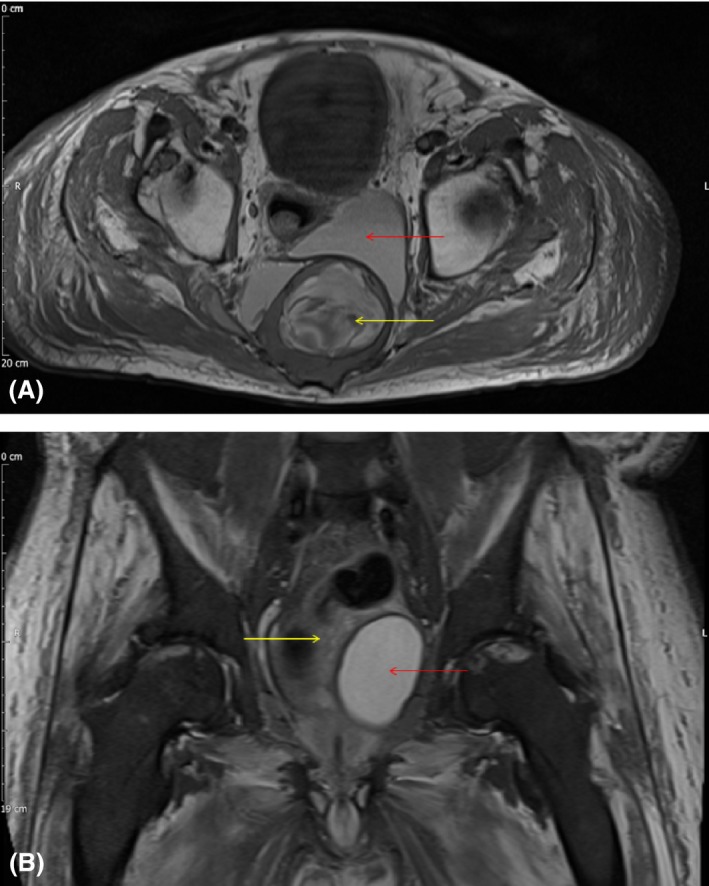
(A and B) T1‐weighted axial magnetic resonance image (A) and T1‐weighted turbo inversion recovery magnitude (TIRM) coronal magnetic resonance image. These demonstrate the large mixed fluid containing (red arrows) and heterogeneous soft tissue (yellow) areas of the presacral pelvic mass consistent with a teratodermoid intimately connected to, and possibly involving the sacrum, with soft tissue extension posterior to the posterior sacral contour.

Ectopic ACTH production from the presacral mass was considered, and a core biopsy of this lesion revealed a well‐differentiated neuroendocrine tumor with no mitoses seen and a Ki‐67 of 1% (WHO grade G1 1). Immunochemistry demonstrated CAM5.2‐positive cytokeratin immunoreactivity and cytoplasmic staining for synaptophysin and ACTH (Fig. 1). Immunostaining for other adenohypophysial hormones (including growth hormone) was negative; however, there was only a small amount of tissue. i(12)p was also negative, in keeping with teratomas arising in this region, and distinguishing this mature sacrococcygeal teratoma from testicular‐type mature teratomas which in adult males have malignant potential 2. Unfortunately, there was insufficient tissue for immunohistochemistry for GHRH. Serum alpha‐fetoprotein and beta‐HCG were normal and he had a normal testicular examination (Fig. 2).

Cushing's syndrome was treated with a combination of subcutaneous octreotide (to reduce ACTH secretion), metyrapone, ketoconazole and, subsequently when eucortisolemia could not be achieved, intravenous etomidate 2.5 mg/h. Once stabilized, he underwent bilateral adrenalectomy with resultant normalization of serum potassium and marked improvement of his edema.

Despite adrenalectomy and good recovery, he continued to experience episodes of hypoglycemia, breathlessness, and anxiety with labile blood pressure. 5‐hydroxyindoleacetic acid (5HIAA) (153 μmol/24 h; reference range 4.7–36.2 μmol/24 h) and chromogranin B (1416 pmol/L; reference range 0–150) were both elevated; chromogranin A was normal. Although the raised 5HIAA was likely responsible for some of these symptoms, they did not completely resolve with low‐dose somatostatin analogue treatment, despite improvement of 5HIAA to 65.3 μmol/24 h, raising the possibility of additional hormonal cosecretion. The most recent ACTH was 47 ng/L (posthydrocortisone), and IGF‐1 has remained normal both prior to, and after, recommencing somatostatin analogue.

## Discussion

Sacrococcygeal teratomas usually show fully differentiated tissue derived from the ectoderm, endoderm, and mesoderm. A very small minority have malignant germ cell elements and may secrete alpha‐fetoprotein or beta‐HCG 3. Most present in utero or in infancy with rectal or bladder obstruction, or rarely weakness, pain, or paralysis 4. A recent study suggests that sacrococcygeal teratomas can be divided into two distinct histological groups, with the presence of i(12)p associated with the presence of germ cell tumor components 5. Treatment is usually surgical with complete resection or, if the teratoma is part of a mixed‐germ cell tumor, adjuvant platinum‐based chemotherapy may be considered.

In this case, core biopsy and immunohistochemistry confirmed the diagnosis of a neuroendocrine tumor within a pre‐existing and well‐differentiated sacrococcygeal teratoma that had been stable since childhood, now with pluripotent neuroendocrine secretion. Surgery would be challenging and would be highly likely to lead to an ileostomy and bladder dysfunction and a significant risk of neurological sequelae. Targeted radiotherapy (to the solid component of the teratoma) has been considered, but has been declined by the patient, and at present, the tumor size remains stable.

Well‐differentiated neuroendocrine tumors arising in testicular teratomas are well recognized and usually behave in an indolent fashion 6. Growth hormone secretion in a mature ovarian teratoma has been previously reported and this remains a possible source for the GH secretion 7. In addition, ovarian teratomas can secrete thyroid hormones from mature thyroid tissue (struma ovarii) leading to thyrotoxicosis in some cases 8, as well as the rarer ovarian carcinoid neoplasms secreting 5HIAA 9. Ectopic GHRH secretion has been observed in human pancreatic tumors 10, and in addition, GHRH has some structural similarity with other peptides secreted within the gut such as VIP, glucagon, and GIP, which are known to weakly stimulate the release of growth hormone from the anterior pituitary 11. However, certain aspects of our case remain to be fully explained. For example, it is notable that there was no sign of pituitary hyperplasia histologically in the sample sent for analysis following transsphenoidal surgery (which might be anticipated in the presence of ectopic GHRH secretion); in addition, unfortunately there was insufficient tissue from the core biopsy of the sacrococcygeal teratoma to permit staining for GHRH (and the patient's IGF‐I was normal at the time of biopsy). It is possible that due to tumor heterogeneity, GHRH or GH expression was present in unsampled areas of the tumor. Nevertheless, on balance, it seems most likely that our patient's neuroendocrine tumor was the origin of his acromegaly.

The cortisol hypersecretion (from ectopic ACTH) was inadequately controlled by medication, necessitating bilateral adrenalectomy. Previous case reports have suggested the existence of ACTH‐dependent Cushing's syndrome in a sacrococcygeal teratoma 12, 13. Our patient is likely to have had a sacrococcygeal teratoma, which was stable for decades, but later in life resulted in acromegaly, then switching to ACTH secretion (resulting in Cushing's syndrome) and serotonin secretion. In addition, it is possible that his current symptoms of breathlessness, hypoglycemia, and fluctuant blood pressure are secondary to secretion of other unidentified peptide(s). To our knowledge, this represents the first reported case of ACTH and serotonin secretion, and possible GHRH or GH cosecretion, from a sacrococcygeal teratoma in an adult.

## Authorship

TB and AMB: involved in the patient's care at the Royal Devon and Exeter Hospital. WMD: involved in the patient's care at St Bartholomew's Hospital. MG: consulted and in charge of methionine PET, EK, and DMB: provided histology reports. TB: prepared the manuscript. All authors contributed to the editing of the manuscript.

## Conflict of Interest

The authors have nothing to disclose.
